# Data on higher education student ethics model

**DOI:** 10.1016/j.dib.2019.104904

**Published:** 2019-11-29

**Authors:** Setyabudi Indartono

**Affiliations:** Yogyakarta State University, Indonesia

**Keywords:** Student ethical behavior, Learning motivation, Student self-efficacy, Resilience, Team strain, Knowledge articulation, Cooperative classroom environment

## Abstract

This article describes data collected between July 2018 and December 2018 in Yogyakarta, Indonesia. The data were collected from 566 Indonesian higher education students who completed a survey. The data were analysed using structural equational modelling (SEM) to develop a model of student ethics.

**Table:** Specification Table

Subject Area	Behaviour
More Specific Subject Area	Student Behaviour
Type of Data	Tables and Figures
How Data were Acquired	1. The data were obtained from 566 students who completed a survey on Indonesian higher education 2. The survey was translated and back-translated from the original to the Indonesian version
Data Format	Raw, analysed, descriptive and statistical data
Experimental Factors	1. Population included higher education students in Indonesia 2. The questionnaire contained data on student ethics, with the following hypothesized constructs: motivation, self-efficacy, resilience, knowledge articulation, team strain, and cooperative classroom environment
Experimental Features	1. Antecedents of student ethics 2. Path model of student ethics
Data Source Location	Yogyakarta State University, Indonesia
Data Accessibility	Data are included in this article

**Table:** 

Value of data • The data from the present sample pertain to the phenomenon of ethical behaviour and represent Indonesian higher education students. • The data will be useful for scholars who are interested in investigating models of ethical behaviour among higher education students in Indonesia. • The datasets can assist in creating comparative models of ethical behaviour of students based on various internal and external pressures. • The data will be valuable for scholars who want to explore comparisons of student ethics inside and outside Indonesian higher education.

## 1 Data

Data were collected from instruments measuring students' ethics, motivation, self-efficacy, resilience, knowledge articulation, team strain, and cooperative classroom environment (Table 1, Table 2, Table 3, Table 4, Table 5, Table 6, Table 7, Table 8). The 14 items from the ethical behaviour (ET1~ ET14) instruments from Rodzalan and Saat were adopted [1]. The 14 items on learning motivation (Mot1~ Mot15) were developed by Mistler-Jackson and Butler Songer [2]. The 6 items on self-efficacy (SE1~ SE6) and the 6 items on resilience (R1~ R6) were developed by Luthans and Youssef [3]. Knowledge articulation (KA1~ KA5) was measured based on 5 items from Kale and Singh [4]. The 3 team strain (TS1~ TS3) items were adopted from Schein [5], and the 5 items on cooperative classroom environment (CCE1~ CCE5) were developed from Premo, Cavagnetto, and Lamb [6].

**Table 1:** Items measuring ethical behaviour.

No	Items
ET 1.	I behave unethically when asked to do so by my lecturers, even though it contradicts my ethical principles.
ET 2.	When my lecturers ask me to do something unethical, I am committed to showing my obedience.
ET 3.	I behave unethically (i.e., plagiarize, stealing) because of pressures (i.e., time and economic constraints).
ET 4.	I prefer not to report friends' unethical behaviour to lecturers.
ET 5.	I commit unethical action when it is beyond my control (e.g., I plagiarize because the academic system emphasises excellent results).
ET 6.	Using a copy machine, paper and other supplies for personal use is not unethical behaviour.
ET 7.	I hold to my principle that honesty is more important than getting good grades.
ET 8.	I take full responsibility for any unethical actions that I take (e.g., I would confess if lecturers found me plagiarizing some assignments).
ET 9.	I behave ethically and adhere to regulations and codes of ethics outlined by the university.
ET 10.	I will accept all opinions/considerations of others if I need to make a decision regarding an ethical dilemma.
ET 11.	During my studies at university, I referred to others to resolve ethical dilemmas.
ET 12.	I personally dealt with ethical dilemmas while studying at university.
ET 13.	I have been confronted with ethical dilemmas during my studies at university.
ET 14.	The faculty (i.e., lecturers, administrators) will reward me when I do something ethical.

**Table 2:** Items measuring learning motivation.

No	Items
Mot 1.	In general, I believe I can do some assignments well, but not all of them.
Mot 2.	In general, I believe I can do any assignment well.
Mot 3.	In general, I believe I can only do a few assignments well.
Mot 4.	In terms of effort, I sometimes try my best.
Mot 5.	In terms of effort, I rarely try my best.
Mot 6.	In terms of effort, I always try my best.
Mot 7.	When my teacher asks a question in class, I volunteer (raise my hand) to answer a lot.
Mot 8.	When my teacher asks a question in class, I never volunteer to answer.
Mot 9.	When my teacher asks a question in class, I volunteer to answer every once in a while.
Mot 10.	If I do not understand something on my homework, the first thing I do is look it up or keep trying by myself.
Mot 11.	If I do not understand something on my homework, the first thing I do is skip it.
Mot 12.	If I do not understand something on my homework, the first thing I do is ask somebody for help.
Mot 13.	I wish my grades were better.
Mot 14.	I am happy with my grades.
Mot 15.	I don't care about my grades.

**Table 3:** Items measuring efficacy.

No	Items
SE1.	I feel confident analysing the long-term problem of finding a solution in my study.
SE2.	I feel confident representing my department at various events.
SE3.	I feel confident contributing to the discussion of learning strategies.
SE4.	I feel confident helping to achieve targets/goals in my department.
SE5.	I feel confident contacting people outside of the department to discuss learning issues.
SE6.	I feel confident presenting information to my study colleagues.

**Table 4:** Items measuring resilience.

No	Items
R1.	When uncertain things happen to me on campus, I usually come to the best conclusion.
R2.	When mistakes happen to me, I take it as a sign of success.
R3.	I always see the positive side of my learning.
R4.	I am optimistic about what will happen to me in the future as relates to my study.
R5.	In achieving my learning goals, I have encountered many failures.
R6.	In learning, I always face various obstacles.

**Table 5:** Items measuring team strain.

No	Items
TS1.	My study group felt that if there was a problem on campus (e.g., grades, communication, etc.), then the course task would help solve those problems.
TS2.	My study group feels that the problems in the campus environment (related to employment opportunities, parent expectations, or curriculum) can be mitigated by course assignments.
TS3.	My study group feels that, if there is a problem with employment, then the industrial practice task can help solve the problem.

**Table 6:** Items measuring knowledge articulation.

No	Items
KA1.	Students involved in various parties are regularly asked about their cooperation experience.
KA2.	Students responsible for cooperation always keep records (in the form of memos, notes, reports, or presentations) of all important activities, decisions or actions related to such cooperation.
KA3.	Students who follow the cooperation programme regularly report progress and performance regarding their respective cooperation.
KA4.	Prodi maintains a ‘repository’ or database containing information from each agency working with it (e.g., date and purpose of establishment of cooperation, name of partners, names of students managing the cooperation, etc.).
KA5.	Prodi has a directory or ‘contact list’ of individuals from within or outside the university who have the potential to provide input or assistance to improve the quality of co-management.

**Table 7:** Items measuring cooperative classroom environment.

No	Items
CCE1.	The class is more fun when I study with other friends.
CCE2.	I prefer to study alone.
CCE3.	I learn best when with my classmates.
CCE4.	I got better grades when I was studying with other friends.
CCE5.	I prefer taking classes where students learn together to solve problems.

**Table 8:** Data collection

Faculty	Response	%
Economic	114	20.1
Engineering	98	17.3
Mathematics and natural science	85	15.0
Social science	61	10.8
Sports science	15	2.7
Art	120	21.2
Educational science	73	12.9
**Total**	566	100.0

## 2 Distribution of students by department

Data were collected from a higher education institution in Indonesia. This study collected 566 surveys completed by respondents from various departments, such as the economic (20.1%), engineering (17.3%), mathematics and natural science (15%), social science (10%), sports science (2.7%), art (21.2%) and educational science (12.9%) departments.

## 3 Data analysis

The dataset was tested for the quality and adequacy of the measurement model, as suggested by Anderson and Garbing [7], to confirm the previous multi-item construct validation, construct validity and construct reliability The deletion of some items was found to increase acceptable fit. The Cronbach's alpha values for each construct [8] are displayed in Table 9, all showing at least 0.7. Thus, internal consistency was found for all of the constructs measured. Convergent validity was determined by the value of the correlation between each construct (Table 10). Fornell and Larcker suggest that correlations lower than .85 among constructs are good [9]. Therefore, the constructs used in this study show good convergent validity.

**Table 9:** Data file items.

Factors	Cronbach α	Items	Loadings
Student Ethics	.844	ET12 ET13	.824 .793
Motivation	.693	Mot5 Mot8 Mot11	.573 .568 .635
Self-efficacy	.825	SE1 SE2 SE3 SE4 SE5 SE6	.541 .585 .622 .596 .609 .685
Resilience	.739	R2 R5 R6	.537 .649 .653
Knowledge articulation	.870	KA1 KA2 KA3 KA4 KA5	.644 .677 .698 .679 .606
Team strain	.912	TS10 TS11 TS12 TS13 TS14 TS15 TS16 TS17	.603 .581 .631 .755 .799 .825 .680 .659
Cooperative classroom environment	.849	CCE1 CCE3 CCE4 CCE5 CCE8 CCE9 CCE10 CCE11	.641 .543 .600 .609 .519 .568 .581 .599

**Table 10:** Correlation among constructs.

		1	2	3	4	5	6	7	8	9	10	11	12	13
1	Sex													
2	Income	.142^b^												
3	Status	0.000	0.000											
4	Faculty	−.133^b^	.026	.014										
5	GPA	−.144^b^	−.174^b^	−.110^b^	−.028									
6	Semester	.100^a^	.012	−.087^a^	−.101^a^	−.054								
7	Ethics	.070	.068	0.000	−.019	−.049	.017							
8	Motivation	.111^b^	.044	.028	−.058	−.138^b^	−.019	.122^b^						
9	Resilience	.055	.069	−.016	−.156^b^	.006	−.020	.114^b^	.127^b^					
10	Self-Efficacy	.045	.008	.010	.008	.057	−.048	.097^a^	−.120^b^	.257^b^				
11	Team Strain	−.112^b^	−.048	.014	.016	.082	−.145^b^	.169^b^	.026	.153^b^	.332^b^			
12	Knowledge Articulation	−.082	−.046	.069	.009	.085^a^	−.061	.150^b^	−.124^b^	.185^b^	.365^b^	.404^b^		
13	Cooperative class environment	−.020	−.044	.068	−.078	.092^a^	−.046	.093^a^	−.027	.150^b^	.348^b^	.330^b^	.363^b^	

a Correlation is significant at the 0.05 level (2-tailed).

b Correlation is significant at the 0.01 level (2-tailed).

## 4 Experimental design, materials and methods

The statistical analysis conducted using AMOS version 7.0 showed that the model had an acceptable fit. The chi-squared test (df = 5, χ^2^ = 28.313) was significant (p < 0.01) [10]. The ratio of chi-square to degree of freedom (df) was 5.66 [11] (CFI = 0.0.947, IFI = 0.948, NFI = 0.938, and TLI = 0.856). Thus, based on the model fit standards endorsed by Marcoulides and Schumacker, the results of CFA indicated a satisfactory fit for the measurement model [12].

**Fig. 1:**
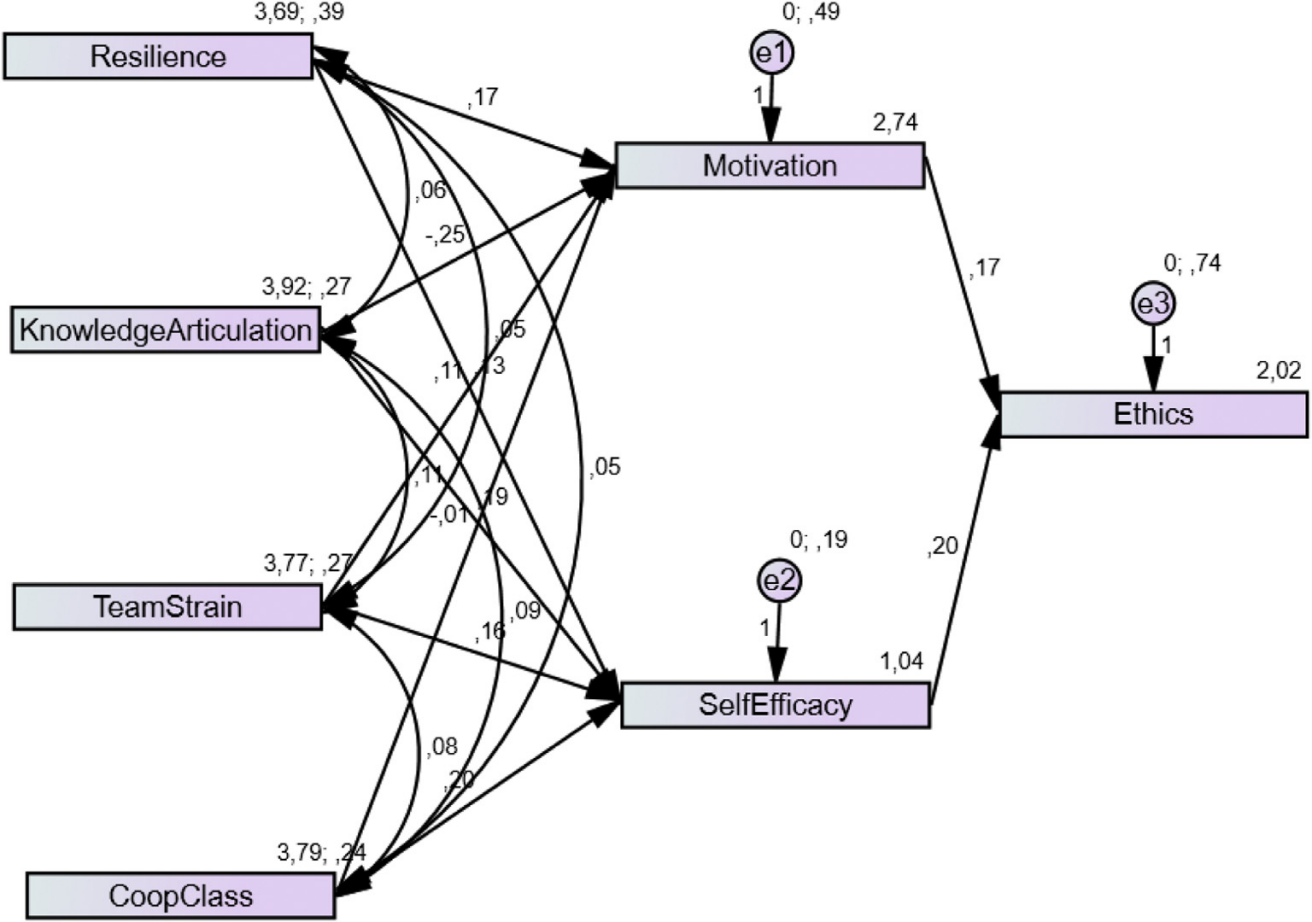
Structural model of student ethical behaviour. Ethics = student ethics, CoopClass = cooperative class environment.

An empirical model testing the effects of motivation, self-efficacy, resilience, knowledge articulation, team strain, and cooperative classroom environment on students’ ethics was examined. The SEM analysis of the final model of the ethical behaviour of higher education students is depicted in Fig. 1. The standardized regression weights of the default model are shown in Table 11.

**Table 11:** Model regression weights.

			Estimate	S.E.	C.R.	P
Motivation	<---	Resilience	0.171	0.048	3.563	***
Motivation	<---	Knowledge Articulation	−0.249	0.065	−3.85	***
Self-Efficacy	<---	Resilience	0.133	0.03	4.403	***
Self-Efficacy	<---	Knowledge Articulation	0.192	0.041	4.699	***
Motivation	<---	Team Strain	0.11	0.064	1.726	0.084
Motivation	<---	Cooperative Class Environment	−0.014	0.066	−0.217	0.829
Self-Efficacy	<---	Team Strain	0.157	0.04	3.912	***
Self-Efficacy	<---	Cooperative Class Environment	0.203	0.041	4.899	***
ethics	<---	Motivation	0.165	0.051	3.267	0.001
ethics	<---	Self-Efficacy	0.196	0.072	2.736	0.006

## 5 Implication of construct modelling

Compared to previous datasets, the validation process of the measurement model [7] included item validity and construct reliability and validity. This behaviour model was used to measure the internal and external factors promoting ethical behaviour among higher education students. According to the fit values of the datasets, further investigations of outcomes are encouraged.
